# Screening with the double surprise question to predict deterioration and death: an explorative study

**DOI:** 10.1186/s12904-019-0503-9

**Published:** 2019-12-27

**Authors:** C. M. M. Veldhoven, N. Nutma, W. De Graaf, H. Schers, C. A. H. H. V. M. Verhagen, K. C. P. Vissers, Y. Engels

**Affiliations:** 1 Department of Anesthesiology, Pain and Palliative Medicine, Radboud University Nijmegen Medical Centre, Nijmegen, the Netherlands; 2 General practice Berg en Dal, Oude Kleefsebaan 96, 6571 BJ Berg en Dal, the Netherlands; 3 Department of Primary and Community Care, Radboud University Nijmegen Medical Centre, Nijmegen, the Netherlands

**Keywords:** Palliative care, General practice, Identification, Prediction, Death

## Abstract

**Background:**

Early identification of palliative patients is challenging. The Surprise Question (SQ1; Would I be surprised if this patient were to die within 12 months?) is widely used to identify palliative patients. However, its predictive value is low. Therefore, we added a second question (SQ2) to SQ1: ‘Would I be surprised if this patient is still alive after 12 months?’ We studied the accuracy of this double surprise question (DSQ) in a general practice.

**Methods:**

We performed a prospective cohort study with retrospective medical record review in a general practice in the eastern part of the Netherlands**.** Two general practitioners (GPs) answered both questions for all 292 patients aged ≥75 years (mean age 84 years).

Primary outcome was 1-year death, secondary outcomes were aspects of palliative care.

**Results:**

SQ1 was answered with ‘no‘ for 161/292 patients. Of these, SQ2 was answered with ‘yes’ in 22 patients. Within 12 months 26 patients died, of whom 24 had been identified with SQ1 (sensitivity: 92%, specificity: 49%). Ten of them were also identified with SQ2 (sensitivity: 42%, specificity: 91%). The latter group had more contacts with their GP and more palliative care aspects were discussed.

**Conclusions:**

The DSQ appears a feasible and easy applicable screening tool in general practice. It is highly effective in predicting patients in high need for palliative care and using it helps to discriminate between patients with different life expectancies and palliative care needs. Further research is necessary to confirm the findings of this study.

## Background

Timely, proactive and multidimensional palliative care has shown its beneficial effects for patients with cancer as well as for those with other life-limiting diseases [1–5]. The majority of people in need of palliative care are of older age [6]. This group will further increase because of an aging population. Moreover, as Bennett and all showed, older age is associated with a shorter duration of palliative care [7].

In the Netherlands, general practitioners (GPs) are important for providing palliative care at home, as they are easily accessible and they often know the patient and his context for years. However, even for GPs it is difficult to identify patients with an increased risk to deteriorate or die and hence might benefit from palliative care. Besides, GPs restrict identification of palliative patients mostly to case-finding [8, 9] and don’t systematically screen their population. As a result, palliative care nowadays mostly remains reactive, terminal care.

To help GPs and other professionals to timely identify patients in need of palliative care, several tools have been developed, [10, 11]. Most of these tools are time-intensive to apply and complex to use. Because they have different indicators per type of disease, it makes them less suitable as a generic screening instrument for daily (general) practice.

One of the instruments however, the Surprise Question (SQ1), is an easy and non-time consuming tool to apply [12]. A clinician asks himself in silence “Would I be surprised if this patient were to die in the next 12 months?” Its accuracy to predict 1-year mortality has been studied in several populations, [13, 14] but the original purpose of the SQ1 is not prognostication but identifying palliative care needs [12, 15]. Unfortunately, its specificity and prognostic accuracy vary largely; a large number of patients identified by the answer ‘no’ on SQ1 are not in need of palliative care, as many are still in an acceptable health condition. Moreover, although desirable, providing structured or specialized palliative care to all patients that are identified by SQ1 when used as a screening tool for a wider population would ask disproportionate time investments and resources.

For these reasons, we developed an additional, second Surprise Question, to be answered when SQ1 is answered with ‘no’: “Would I be surprised if this patient will be still alive after 12 months?” (SQ2). We hypothesized that adding SQ2 if SQ1 is answered with ‘no’ helps to select those patients with a high chance of deterioration or dying within 1 year, and thus are in urgent need of early palliative care. In two case vignette studies GPs considered the combination of SQ1 and SQ2, called the double SQ (DSQ), a useful tool and it triggered them to plan more anticipatory, multidimensional palliative care for those they considered most vulnerable [16, 17].

However, the DSQ has not been studied in a prospective study. In this explorative study we therefore compared the accuracy (sensitivity, specificity and predictive values) of screening patients ≥75 years in general practice with the DSQ regarding 1-year mortality to SQ1 alone, and compared health care needs and actually provided palliative care in relation to the answers on the DSQ.

## Methods

### Design

We performed an explorative, prospective study with a retrospective medical record review. In May 2016, two GPs (CV and WG) answered the DSQ for each included patient of their dual practice.

### Participants and setting

Patients were not involved in the design of the study. Participants were two GPs in a dual practice in the Southeastern part of the Netherlands. In this practice, both GPs often have a longstanding relationship with their patients. One male GP (CV; 57 years of age, 21 years of experience) had had specialized training in palliative care, the other female GP (WG; 42 years of age, 13 years of experience as a GP) had had specialized training in elderly care.

### Procedure

In 2016, both GPs together, in consensus, answered SQ1 for each patient on their patient list aged 75 years or older. If SQ1 was answered with ‘no’, they answered SQ2. By restricting identification to this age category, time investment was feasible, while selecting the majority of patients at risk of deteriorating or dying. In the Netherlands, in 2016 the mean age at death was 75.6 years for men and 80.5 years for women; 66% of the population dies at the age of 75 years or older [6]. No exclusion criteria were used. The answers were kept in a sealed envelope, not documented in the patient files and not used while planning care for patients in the following year.

### Ethics

The study was approved by the research ethics committee of the Radboud University Nijmegen Medical Center, case number 2017–3552. In this academic GP practice, all patients have been informed that data from their medical record may be used for research; if they don’t want their data to be used for this, they can opt out. Anonymity was guaranteed. The researcher was, in her role as medical student, part of the general practice team. In the Dutch law, written informed consent for medical record review is not required.

### Outcome measures

The primary outcome was death at 12 months.

Next, we related the answers to the SQs to the quantity of received palliative care (secondary outcome measures 1 and 2). Besides, based on the WHO definition of palliative care that states that it should be multidimensional and proactive, [18] secondary outcome measures 3 and 4 were chosen to measure the quality of palliative care:

1. Number of consultations with the GP practice (consultations at practice, home visits, telephone contacts, consultations with the practice assistant and consultations with the practice nurse);
2. Number of contacts with the out of hours GP cooperation, emergency room (ER) visits and hospitalizations;
3. Quality of palliative care and advance care planning (ACP). To analyze these aspects, we checked which palliative domains (somatic, social, psychological and spiritual) and patient preferences for treatment and end-of-life care (ACP directives) were discussed and documented.
4. Place of death

### Data collection

One year after the SQs had been answered, the medical records of all screened patients were blindly reviewed by an independent researcher (NN) who did not know the answers to the SQs.

Characteristics of all screened patients of whom medical records were available were retrieved and also analyzed per answering category group (Table 1). Of the patients that had moved and left the practice in the year after the SQs were answered, only eventual date of death was considered; for them, the secondary research questions were not answered.

One researcher (NN) extracted data from the medical records according to a case report form (Table 2). In case of doubt issues were discussed with a GP (CV) for clarification of what was written and with a non-clinical researcher (YE) for interpretation whether something occurred or didn’t occur. As a double check, the 15 files that had firstly been analyzed were re-analyzed at the end.

**Table 1:** Characteristics of patients aged ≥75 years, grouped by SQ1 answers and DSQ answers

	SQ1		DSQ			
	Group 1 (SQ1 yes) *n* = 131^a^	Group 2 (SQ1 no) *n* = 161	Group 1 (SQ1 yes) *N* = 131^a^	Group 2a (SQ1 no, SQ2 no) *n* = 139	Group 2b (SQ1 no, SQ2 yes) *n* = 22	All patients *n* = 292
Age; mean (sd)	82 (4.67)	86 (5.24)	82 (4.67)	86 (5.23)	87 (5.32)	84 (5.46)
Gender; female n (%)	87 (131 (66%)	88/161 (55%)	87 (131 (66%)	77/139 (55%)	11/22 (50%)	175/292 (60%)
Living situation; n (%)						
-at home	88/122 (72%)	91/154 (59%)	88/122 (72%)	79/133 (59%)	12/21 (57%)	179/276 (65%)
-residential home	33/122 (27%)	61/154 (40%)	33/122 (27%)	52/133 (39%)	9/21 (43%)	94/271 (34%)
-with family, other than partner	1/122 (1%)	2/154 (1%)	1/122 (1%)	2/133 (2%)	0/21 (0%)	3/276 (1%)
Marital status; n (%)						
-married or living together	69/124 (56%)	55/156 (35%)	69/124 (56%)	48/135 (36%)	7/21 (33%)	124/280 (44%)
-widow or single	55/124 (%)	101/156 (65%)	55/124 (44%)	87/135 (64%)	14/21 (67%)	156/280 (56%)
Care provider; n (%)						
-child	19/123 (15%)	65/155 (42%)	19/123 (15%)	55/134 (41%)	10/21 (48%)	84/278 (30%)
-partner	1/123 (1%)	6/155 (4%)	1/123 (1%)	6/134 (5%)	0/21 (0%)	7/278 (3%)
-other	10/123 (8%)	18/155 (12%)	10/123 (8%)	14/134 (10%)	4/21 (19%)	28/278 (10%)
-unknown or none	93/123 (76%)	66/155 (43%)	93/123 (76%)	59/134 (44%)	7/21 (33%)	159/278 (57%)
Home care; n (%)	18/123 (15%)	83/155 (54%)	18/123 (15%)	67/134 (50%)	16/21 (76%)	101/278 (36%)
Diseases^b^; n (%)						
-cancer	2/123 (2%)	19/155 (12%)	2/123 (2%)	11/134 (8%)	8/21 (38%)	21/278 (8%)
-organ failure^c^	24/123 (20%)	38/155 (25%)	24/123 (20%)	67/134 (50%)	16/21 (76%)	107/278 (39%)
-cognitive impairment	10/123 (8%)	38/155 (25%)	10/123 (8%)	34/134 (25%)	4/21 (19%)	48/278 (17%)
-chronic heart failure	7/123 (6%)	23/155 (15%)	7/123 (6%)	15/134 (11%)	8/21 (38%)	30/278 (11%)

^a^For some patients that have moved out of the practice, data are missing and therefore totals differ

^b^Patients were often diagnosed with more diseases, therefore total percentages exceed 100%

^c^Organ failure: defined as having COPD, chronic heart failure, chronic kidney failure and/or neurological degenerative disease

**Table 2:** Format for extracting data out of the patient files

Consultations GP	Consultations at practice, also for small surgery.
Telephonic consultations GP	Telephonic consultations or mail contact with patient or caregiver/family, not regarding practicalities such as faxing medical data, or only to inform about lab results. Also: A reaction from the GP at a medical question from the patient, asked by telephone to the assistant.
Home visits GP	Home visits of the GP.
Consultations practice nurse	Consultations, telephonic consultations, home visits, mail contact and reactions to questions from the patient asked to the assistant, by the somatic and psychological practice nurse or the specialized nurse, not regarding practicalities, or only to inform about lab results.
Consultations practice assistant	Consultations, telephonic consultations, home visits, mail contact and reactions to questions from the patient, not regarding practicalities such as faxing medical data or to repeat medication recipes, or only to inform about lab results. Also for diagnostic tests, wound controls and small surgery. If the (telephonic) consultation of the assistant was followed by a consultation or home visit of the GP, only the latter was counted.
Quality of palliative care and ACP	Everything regarding the aspects of palliative care and ACP, noted in the patient file by the GP, the assistant, the practice nurse or the specialized nurse.
	If a will statement was uploaded into the patient file, any ACP directives that it contained were counted.
	Dimensions:
	Somatic: symptoms, complaints, general health
	Psychological: fear, depressed mood, emotions, anger, denial, anxiety, worries
	Social: social contacts, tensions between patient and loved ones or care providers, financial worries, leaving loved ones when dying, saying goodbyes
	Existential: things that occupy someone, balance of life, questions regarding life and death, preparing for dying, a wish to die, feeling powerless or dependant, hope, faith
Home care	If anywhere during the year, home care was given, it was counted as ‘yes’.
Diseases	Also diseases that were diagnosed before the screening with the SQs were counted.
	Cancer: only when active disease or active treatment
	Cardiovascular disease: all cardiovascular diseases, including hypertension, cardiovascular incidents and vascular disease.

Characteristics or outcomes not mentioned here were unambiguous to extract

**Table 3:** Accuracy of SQ1 and of SQ2

	Total, n	Died, n	Survived, n	Sensitivity^a^, % (95% CI)	Specificity^a^, % (95% CI)	PPV^a^, % (95% CI)	NPV^a^, % (95% CI)
SQ1							
No	161	24	137	24/26 = 92.3% (74.9–9.1%)	129/266 = 48.5% (42.4–54.7%)	24/161 = 14.9% (9.8–21.4%)	129/131 = 98.5% (94.6–99.8%)
Yes	131	2	129				
Total	292	26	266				
SQ2^b^							
Yes	22	10	12	10/24 = 41.7% (22.1–3.4%)	125/137 = 91.2% (85.2–95.4%)	10/22 = 45.5% (24.4–67.8%)	125/139 = 89.9% (83.7–94.4%)
No	139	14	125				
Total	161	24	137				

Specificity: ability to correctly detect patients who are not dying

*PPV* Positive predictive value: ability to predict death

*NPV* Negative predictive value: ability to predict survival

^a^ Sensitivity: ability to correctly detect patients who are dying

^b^ SQ2 was only answered for patients with a negative answer to SQ1

**Table 4:** Contact with GP and out of hours service, ER visits and hospitalizations; means and standard deviations per answering group on the DSQ

	Group 1 (*n* = 122)^a^	Group 2 (*n* = 129)^b^	Group 3 (*n* = 21)^c^
Consultations GP; mean (SD)			
- At practice	3.45 (2.81)	3.69 (3.36)	3.90 (4.73)
- Telephonic consultation	1.58 (1.66)	2.99 (3.67)	2.05 (1.53)
- Home visits	1.10 (2.39)	4.46 (6.79)	7.10 (6.72)
Total	6.13 (4.42)	11.14 (8.29)	13.05 (6.98)
Total contact with general practice^d^	8.87 (6.11)	15.23 (10.49)	17.24 (9.16)
Contact with out of hours service; mean (SD)			
- Consultation	0.05 (0.22)	0.09 (0.35)	0.00 (0.00)
- Telephonic consultation	0.11 (0.38)	0.37 (0.88)	0.81 (1.12)
- Home visit	0.09 (0.43)	0.31 (0.71)	0.48 (0.81)
Total	0.25 (0.68)	0.77 (1.42)	1.29 (1.59)
Emergency Room visits; mean (SD)	0.19 (0.50)	0.26 (0.55)	0.14 (0.36)
Hospitalizations; mean (SD)	0.15 (0.42)	0.16 (0.42)	0.24 (0.54)

^a^Group 1: SQ1 = yes

^b^Group 2: SQ1 = no, SQ2 = no

^c^Group 3: SQ1 = no, SQ2 = yes

^d^Also including contacts with GP practice assistant and practice nurse

**Table 5:** Palliative care aspects, analyzed per answering group on the DSQ

	Group 1 (*n* = 122)^a^	Group 2 (*n* = 129)^b^	Group 3 (*n* = 21)^c^
Dimensions discussed^d^; n (%)			
- Somatic	115 (94%)	127 (98%)	21 (100%)
- Social	32 (26%)	49 (38%)	8 (38%)
- Psychological	45 (37%)	59 (46%)	12 (57%)
- Existential	22 (18%)	41 (32%)	12 (57%)
Total number of dimensions; median (IQR)	2 (1)	2 (2)	3 (1)
ACP directives^e^; median (IQR)	0 (0)	0 (0)	0 (2)
ACP aspects^d^; n (%)			
- Discussing end-of-life wishes	12 (10%)	17 (13%)	3 (14%)
- Discussing dying scenarios	0 (0%)	2 (2%)	3 (14%)
- Discussing preferred place of death	0 (0%)	4 (3%)	4 (19%)
- Assignment for out of hours GP care	5 (4%)	16 (12%)	5 (24%)
Total number of ACP aspects and directives; median (IQR)	0 (0)	0 (1)	1 (3)
At least one ACP aspect or directive	24 (20%)	44 (34%)	11 (52%)
At least one ACP aspects or directive discussed before May 2016	41 (34%)	75 (58%)	17 (81%)
At least one ACP aspects or directive, over all time	53 (43%)	91 (71%)	19 (90%)
Other palliative care aspects^a^; n (%)			
- Discussing personal aspects of quality of life	0 (0%)	3 (2%)	0 (0%)
- Discussing personal goals	2 (2%)	4 (3%)	3 (14%)
- Discussing preferences for treatment	18 (15%)	42 (33%)	5 (24%)
- Involving family and loved-ones in planning care	18 (15%)	42 (33%)	11 (52%)
- Providing care for family and loved-ones	2 (2%)	10 (8%)	3 (14%)

^a^Group 1: SQ1 = yes

^b^Group 2: SQ1 = no, SQ2 = no

^c^Group 3: SQ1 = no, SQ2 = yes

^d^Dimensions, ACP aspects and palliative care aspects: when at least once discussed and documented

^e^Directives include: CPR, hospital admissions, mechanical ventilation, antibiotics and artificial feeding and liquid administration

Of all included patients, characteristics were described (age, gender, living situation, marital status, whether receiving home care, types of diseases and whether the patient died in the 12 months after screening. For all secondary outcomes, the medical records were retrospectively analyzed for the period of 1 year after the screening. As advance care planning (ACP) could already have been performed before screening took place, we also checked the medical records on end of life preferences of the period before the screening.

Data were kept in Castor, a valid database that meets the Good Clinical Practice criteria. After all data had been extracted, the database was locked. Next, data were exported to SPSS, where the answers to the SQs were added.

### Analyses

Statistical analyses were performed with IBM SPSS software version 22. Based on the answers to the SQs, patients were grouped in three possible answer combination groups (group 1: SQ1 answered with ‘yes’; group 2: SQ1 ‘no’, SQ2 ‘no’; group 3: SQ1 ‘no’, SQ2 ‘yes’). Descriptive statistics were used to describe characteristics of all patients and of the patients per group.

To answer the primary outcome, we related the answers to both SQs to 1-year mortality and calculated sensitivity, specificity, positive and negative predictive value (PPV and NPV) of SQ1 and of SQ2. Sensitivity (ability to correctly identify patients who will die) and specificity (ability to correctly identify patients who will not die) were calculated, as well as the positive and negative predictive value (PPV and NPV: ability to respectively predict death and survival) regarding 1 year mortality for respectively SQ1 and SQ2. For each group, frequencies, means and standard deviations of the secondary outcome variables were calculated with descriptive statistics. Besides, for all secondary outcomes we analyzed if there were differences between the deceased patients with different answers to the SQs.

## Results

### Patients

**Fig. 1:**
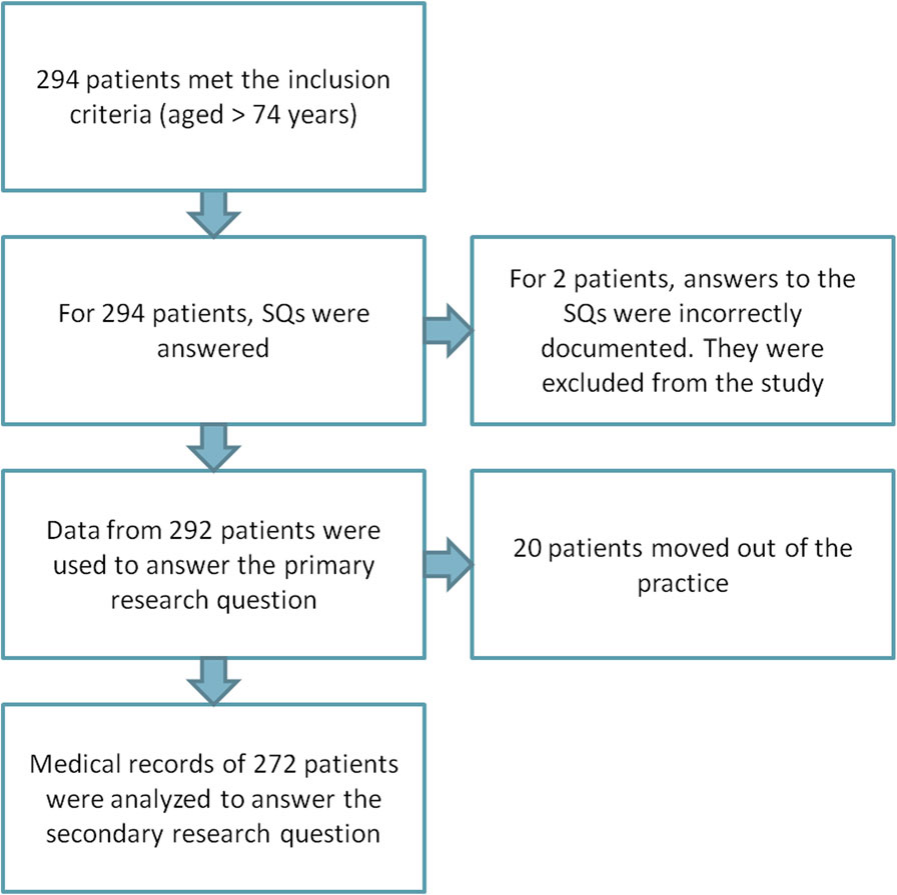
Flow chart: Inclusion and analysis of patients

At the day of screening, 294 (15%) of the 1960 patients on the patient list were aged 75 years or older, and for 292 of them, SQ1 and SQ2 were answered by the two GPs, which took them about 3 h in total. One year later 20 patients had moved and were no longer on the patient list of the practice, and, according to the Dutch privacy law, their medical records had become inaccessible. However, information on their survival could be retrieved. Therefore, primary outcomes were based on data of 292 patients, and secondary outcomes on data of 272 patients (Fig. 1).

For 131 patients, the answer to SQ1 was ‘yes’; the GP would be surprised if these patients would die within a year (group 1). These patients had a lower mean age, more often lived at home and had less morbidities, with exception of cardiovascular disease, than patients for whom SQ1 was answered with ‘no’. SQ1 was answered with ‘no’ for the remaining 161 patients: Of these, SQ2 was answered with “no” in 139 patients (the GP would not be surprised if they would be still alive; group 2) and with ‘yes’ in 22 patients (the GP would be surprised if they would be still alive; group 3). Compared to the patients in groups 1 and 2, patients in group 3 more often received home care and more often were diagnosed with cancer or organ failure.

### Deaths – primary outcome

In total 26 patients died during the year of study, of whom two, who both died unexpectedly, had not been identified with SQ1 (SQ1 = no) (sensitivity SQ1: 92%). The specificity of SQ1 was 49%. Of the 161 patients identified with SQ1, 24 died (PPV SQ1: 15%). The negative predictive value (NPV) of SQ1 was 99% (Table 3).

Ten of the deceased patients had been identified with SQ2 (SQ2 = yes), 14 had not (sensitivity SQ2: 42%). The specificity of SQ2 was 91%. With SQ2, 22 patients had been identified, of whom ten had died (PPV SQ2: 45%). The NPV of SQ2 was 90% (Table 3).

After a year, 46% of the patients in group 3 had died, compared to 10% of the patients in group 2 and 2% of the patients in group 1.

### Secondary outcomes

#### Consultations with GP

The mean number of contacts (all types) with the GP was lowest for group 1 and highest for group 3 (group 1: 6.13; group 2: 11.14; group 3: 13.05).

#### Out of hours GP cooperation, ER visits, hospitalizations

The mean number of contacts with the out of hours service was 0.25 in group 1, 0.77 in group 2 and 1.29 in group 3. No large differences in number of ER visits and hospitalizations were found (Table 4).

#### Palliative care provision

Regarding the documentation of palliative care aspects, figures were almost always highest in group 3 and lowest in group 1 (Table 5). Existential issues were discussed with 57% of the patients in group 3, with 32% of the patients in group 2 and with only 18% of the patients in group 1. Advance care planning was done most often in group 3 (52% versus 34% in group 2 and 20% in group 1) and in the majority of the patients in group 3 (81%), advance care planning often had already been started before the screening with the SQs.

## Discussion

### Summary

In this study, we investigated the outcome of the Double Surprise Question (DSQ): adding SQ2 to SQ1 if SQ1 is answered with ‘no’, as a proactive screening tool for palliative care needs in primary care. We found a low specificity of SQ1, meaning that many patients were incorrectly identified. For SQ2, we found a low sensitivity, which means that more patients were missed. Therefore each of both SQs on its own is inaccurate in predicting death. However, by asking both questions, a division into three groups was made with largely different death rates (highest in group 3 (SQ1: no, SQ2: yes) and lowest in group 1 (SQ1: yes). Furthermore, patients in group 3 had more contacts with the GP and the out of hours GP service, and aspects of palliative care and advance care planning were more often discussed with these patients than with patients in groups 1 and 2. Patients in group 1 had the lowest figures for these outcomes.

Our findings suggest that the DSQ discriminates between patients with different life expectancies and care consumptions, and also show that SQ2 complements SQ1. The differences in provided care between the two answering categories of SQ2 (group 2 versus group 3), but also between the two categories of SQ1 (group 1 versus groups 2 plus 3 together), show that SQ2 cannot replace SQ1; SQ1 and SQ2 are meant to be applied together.

Because of the clear differences between the three groups, the answers to the DSQ seem related to palliative care needs, although we realize that care needs are not equal to care consumption. In general practice, it is not feasible to provide proactive palliative care to all patients identified with SQ, because of time constraints. Moreover, providing proactive palliative care to patients not in need of it is undesirable for patients and GPs.. SQ2 however divides those patients selected with SQ1 in a small group to focus proactive palliative care on, and a larger group to monitor less intensively.

### Strengths and limitations

The DSQ is a simple, innovative, and easy applicable screening tool with the potential to predict individual death and palliative care needs of older patients in daily general practice. It offers the possibility to improve the correct identification of palliative patients. With our study design, we were able to gain information about its properties, taking other outcomes than 1-year mortality in account. While planning care for their patients, the GPs were therefore not actively influenced by the answers to the SQs, although there is a chance that the GPs could recall the small number of patients in group 3 which might have influenced the results. However, if this was the case, to our opinion this recalling of the patients in group 3 will be more related to the frail condition of these patients than to the answers to the SQs. In this prospective study we were able to screen in the whole population elderly, with a high prevalence of death and palliative care.

However, this study has also some limitations. This explorative study was performed in one general practice, where both GPs have been extensively trained in respectively palliative care and frail elderly care and are familiar with both SQs, and are both authors of this paper. This might have influenced the findings.

Next, we studied the DSQ in patients aged 75 and older, implying that we have no information on its value for younger patients.

Almost half of the patients in group 3 died during the year. This means that they had less time to have had contacts with the GP. If they had lived the entire year, they probably would have had more contacts. This may have led to an underestimation of the quantity and also quality of care in group 3.

### Comparison with existing literature

Over the past decade, studies on the original SQ (SQ1) in different settings and in different patient groups were always limited to prognostication of death. However, palliative care needs are not always linked to prognosis. The original aim of SQ1 was to identify if a patient is thought to benefit from palliative care services [12] and not to determine if a patient is likely to die in the next year. Our study is the first to study other proxies for palliative care needs as well. Next, up to now the value of SQ1 has been mainly studied in hospital settings or in patient groups with specific, advanced diseases. The only exception is a study of Mitchell et al., who also studied SQ1 as a screening tool in elderly patients in general practice [19]. Finally, this is the first time that the DSQ has been studied prospectively.

Recent meta-analyses showed large ranges in sensitivity and specificity of the individual SQ1 for different studies in homogeneous populations [13, 14]. However, in a daily clinical primary care practice, both high sensitivity and a high specificity are needed to identify the individual patients with a high certitude to die in the coming year, in order to adapt proper care planning in the high through put practice of the future.

Some criticisms at the use of SQ1 as a screening tool have been expressed [20–22]. Because of its moderate predictive values for death, the limited research in patients with non-cancer disease and the lack of evidence that a negative answer to SQ1 correlates with palliative care needs, resistance against its prominent position in extensive screening tools for palliative needs and in palliative care guidelines have been raised. Within this study, we screened all elderly and included other proxies for palliative care needs, thereby providing more information about the properties of SQ1, and also of SQ2.

## Conclusions

The DSQ is an innovative, easy and fast screening tool with the potential to predict individual death and palliative care needs of (older) patients in daily general practice as it divides the patient population into three groups with different life expectancy and care consumptions. Further research is necessary to confirm the findings in this explorative study.

Using the DSQ is not meant to predict survival, but to trigger GPs to think about the condition of the patient and to use this awareness while planning and providing palliative care [1, 23, 24].

## Data Availability

Data are available upon request via the corresponding author.

## Abbreviations

ACP: Advance Care Planning
DSQ: The Double Surprise Question
ER: Emergency Room
GP(s): General Practitioner(s)
NPV: Negative Predictive Value
PPV: Positive Predictive Value
SQ1: The Surprise Question
SQ2: The second Surprise Question
SQs: Surprise Questions
WHO: World Health Organization
